# Exercise training enhances *in vivo* clearance of endotoxin and attenuates inflammatory responses by potentiating Kupffer cell phagocytosis

**DOI:** 10.1038/s41598-017-12358-8

**Published:** 2017-09-20

**Authors:** Shoichi Komine, Kentaro Akiyama, Eiji Warabi, Sechang Oh, Keisuke Kuga, Kazunori Ishige, Shinji Togashi, Toru Yanagawa, Junichi Shoda

**Affiliations:** 1 Graduate School of Comprehensive Human Sciences, University of Tsukuba, Ibaraki, 305-8575 Japan; 2 The Center of Sports Medicine and Health Sciences, Tsukuba University Hospital, Ibaraki, 305-8576 Japan; 3 Japan Society for the Promotion of Science, Tokyo, 102-0083 Japan; 4 Division of Molecular and Cellular Physiology, Faculty of Medicine, University of Tsukuba, Tsukuba, Ibaraki, 305-8575 Japan; 5 Medical Sciences, Faculty of Medicine, University of Tsukuba, Tsukuba, Ibaraki, 305-8575 Japan; 6 Division of Cardiology, Faculty of Medicine, University of Tsukuba, Tsukuba, Ibaraki, 305-8575 Japan; 7 Division of Gastroenterology, Faculty of Medicine, University of Tsukuba, Tsukuba, Ibaraki, 305-8575 Japan; 8 Division of Plastic Surgery, Faculty of Medicine, University of Tsukuba, Tsukuba, Ibaraki, 305-8575 Japan; 9 Division of Oral and Maxillofacial Surgery, Faculty of Medicine, University of Tsukuba, Tsukuba, Ibaraki, 305-8575 Japan; 10 Plastic Surgery, Shonai Amarume Hospital, Yamagata, 999-7782 Japan

## Abstract

The failure of Kupffer cells (KCs) to remove endotoxin is an important factor in the pathogenesis of non-alcoholic fatty liver disease (NAFLD). In this study, the effects of exercise training on KC function were studied in terms of *in vivo* endotoxin clearance and inflammatory responses. Mice were allocated into rest and exercise groups. KC bead phagocytic capacity and plasma steroid hormone levels were determined following exercise training. Endotoxin and inflammatory cytokine levels in plasma were determined over time following endotoxin injection. KC bead phagocytic capacity was potentiated and clearance of exogenously-injected endotoxin was increased in the exercise group. Inflammatory cytokine (TNF-α and IL-6) levels were lower in the exercise group. We found that only DHEA was increased in the plasma of the exercise group. In an *in vitro* experiment, the addition of DHEA to RAW264.7 cells increased bead phagocytic capacity and attenuated endotoxin-induced inflammatory responses. These results suggest that exercise training modulates *in vivo* endotoxin clearance and inflammatory responses in association with increased DHEA production. These exercise-induced changes in KC capacity may contribute to a slowing of disease progression in NAFLD patients.

## Introduction

The problem of obesity continues to worsen as a result of several factors including the westernization of dietary habits and chronic insufficient exercise. According to a recent Japanese nationwide survey, the prevalence of abnormal hepatic function in adults is rapidly increasing^1^. The increased incidence of non-alcoholic fatty liver disease (NAFLD) contributes significantly to this trend. In some cases, NAFLD progresses to non-alcoholic steatohepatitis (NASH), which is progressive and eventually leads to hepatic cirrhosis or liver cancer.

The “multiple parallel hits” hypothesis has been applied to the pathogenesis of NAFLD. In this context, it is suggested that several factors, including insulin resistance, adipokines, oxidative stress, and enteric bacteria, all damage the liver^2^. Endotoxin, an enteric bacterially-derived molecule, is thought to play an important role in NAFLD pathogenesis^3^. In patients with NASH, intestinal permeability is enhanced in fragile intestinal epithelium such that endotoxin passes more easily into the portal vein, resulting in endotoxemia^4^. Excessive endotoxin is recognized by Toll-like receptors (TLRs) on Kupffer cells (KCs), thereby triggering the inflammatory response. This may lead to accumulation of fat in the liver, development of fibrosis, and eventually NASH. Thus, the capacity of KCs to remove endotoxin may be important in attenuation of the progression of NAFLD.

KCs are liver-resident macrophages, accounting for approximately 80% of the macrophages in the whole body^5^. KCs participate in the initial step of the innate immune response: phagocytosis of harmful exogenous substances such as endotoxin^6,7^. Phagocytic capacity is known to be regulated by hormones and cytokines^8^. In NAFLD, reduced phagocytic capacity of KCs has been reported both in humans and in animal models^9–11^. Phagocytic capacity is reduced in parallel with progression of hepatic lesions in NAFLD^9^. We developed a mouse model of NASH, the *p62:Nrf2* double knockout mouse^12^ that exhibited reduction in KC phagocytic capacity. In our model, endotoxaemia and the activated KC inflammatory response, both associated with the KCs dysfunction, led to chronic inflammation in the liver. This suggests that abnormalities in KC phagocytic capacity may cause a reduction in *in vivo* endotoxin clearance, leading to progression of inflammatory liver disease. These conditions are assumed to be involved in the aggravation of NAFLD hepatic lesions and the progression from simple steatosis to NASH.

Prevention and treatment of NAFLD present important medical challenges in clinical practice. At present, there is no consensus regarding prevention and treatment of NAFLD apart from diet and exercise therapy. Furthermore, there is insufficient evidence in support of exercise therapy as an effective modality, compared with diet therapy.

A meta-analysis suggested that exercise therapy may be useful for improvement of hepatic lipid accumulation in NAFLD^13^. Our group performed a clinical study of exercise therapy suggesting that increased physical activity at moderate to high intensity decreased hepatic lipid accumulation and ameliorated inflammatory/oxidative stress^14^. In this randomized comparison study, hepatic lipid accumulation and hepatic stiffness was reduced in the high-intensity aerobic exercise group compared with the resistance exercise group. The high-intensity aerobic exercise group also demonstrated improved foreign-body phagocytosis by KCs^15^. Recovery from suppression of KC activity may contribute to these exercise-induced improvements in NAFLD-related changes.

Acute exercise in animal models affects levels of steroid hormones^16,17^ and increases KC bead phagocytic capacity^16^. These results suggest that exercise may modulate KC function such as *in vivo* clearance and the inflammatory response to endotoxin. However, the specific effects of exercise on KCs and the details of the molecular mechanisms in NAFLD have yet to be elucidated.

In this study, we investigated the effects of exercise training on KC function, particularly on foreign-body phagocytic capacity, *in vivo* endotoxin clearance, and the inflammatory response to endotoxin. We also performed an *in vitro* study of the effects of steroid hormones on bead phagocytic capacity and the inflammatory response to endotoxin in RAW264.7 cells.

## Methods

### Animal care

Seven-week-old male C57/BL/6 J mice were obtained from Charles River Japan (Kanagawa, Japan). The mice were housed with a 12-h light/dark cycle, at room temperature 23.5 ± 2.5 °C, and humidity at 52.5 ± 12.5%. The mice had *ad libitum* access to food and water. After preliminary breeding for 1 week, the mice were randomly divided into two groups: 3-month rest group (3moR), and 3-month exercise group (3moTr). For the exercise group, treadmill exercise was undertaken (MK-680, Muromachi Kikai). Exercise groups were habituated to treadmill running for 3 days. First day intensity for adaptation was 5 m/min for 10 min and 10 m/min for 10 min. Second and final day intensity was 10 m/min for 10 min and 15 m/min for 10 min. Going forward, exercise intensity was increased to 10 m/min for 5 min, 12 m/min for 5 min, 14 m/min for 5 min, 16 m/min for 5 min, and 18 m/min for 30 min, for a total of 50 min once per day, 5 times per week. To generate macrophage-deficient mice following the 3-month period, we injected Clophosome^®^-A (FormuMax Scientific) at 8 µL/g body weight immediately following the exercise load. We identified this group as the 3-month rest group with Clophosome-A (Lipo3moR) and the 3-month exercise group with Clophosome-A (Lipo3moTr).

Animal experiments were carried out humanely in accordance with the Regulations for Animal Experiments of University of Tsukuba, and the Fundamental Guidelines for Proper Conduct of Animal Experiments and Related Activities in Academic Research Institutions under the jurisdiction of the Ministry of Education, Culture, Sports, Science, and Technology of Japan, and with approval from the Institutional Animal Care and Use Committees of University of Tsukuba.

### Sample collections

Following the exercise protocol, the mice rested for 1 day and were anaesthetized with an intraperitoneal injection of pentobarbital (50 mg/kg body weight). We measured body weight and wet weight of the following: liver, epididymal fat, muscles (soleus, gastrocnemius, plantaris, and tibialis anterior). Samples were then rapidly frozen. Blood samples were collected from the inferior vena cava and centrifuged at 4000 rpm at 4 °C for 15 min. Tissue samples and supernatant were stored at −80 °C. For estimation of whole body skeletal muscle mass, visceral fat mass, and subcutaneous fat mass, anesthetized mice were placed on a bed made of polystyrene form and scanned by a micro-CT scanner (Latheta, LCT-200, Hitachi Aloka Medical).

### Separation of liver non-parenchymal cells and analysis of Kupffer cell foreign-body phagocytosis

For the KC phagocytosis assay, we isolated non-parenchymal cells from livers one day following exercise. The mice were anesthetized and perfused with liver perfusion medium (Gibco) for 4 min, followed by DMEM with collagenase Type 4 (Worthington Biochemical), trypsin inhibitor (Wako Chemical), and HEPES (Dojindo) at 37 °C for 10 min. Liver samples were collected and cells were isolated in hepatocyte wash medium (Gibco). To remove parenchymal cells, the samples were centrifuged three times at 30 g for 2 min. The supernatants were centrifuged at 400 g for 8 min, and precipitates including KCs were obtained. The samples were analysed by flow cytometry.

### KC foreign-body phagocytosis analysed by flow cytometry

Prior to 5 min of liver perfusion, latex beads (Invitrogen, FluoSpheres^®^, 1.0 µm diameter, carboxylate-modified) at 0.57 µl/g body weight were injected in the tail vein. Following liver perfusion, the cells were isolated. KC surface marker F4/80 was stained. Percentage of phagocytic cells and fluorescent intensity of latex beads in F4/80-positive cells were evaluated by flow cytometry.

The expression levels of KC surface proteins involved in phagocytosis (CD68, macrophage receptor with collagenous structure; MARCO, macrophage class A scavenger receptors; SR-A) were analysed by flow cytometry. Samples were incubated with APC-conjugated anti-F4/80 (17-4801-82, eBioscience), PerCP/Cy5.5- conjugated anti-CD68 (137010, BioLegend), MARCO (MCA1849, Bio-Rad) and SR-A (AF1797, R&D Systems), followed by incubation with the secondary antibody Alexa Fluor 488 (Invitrogen). All flow cytometry data were collected on a Gallios flow cytometer (Beckman Coulter). Data were analysed using Kaluza flow cytometry data analysis software (v. 1.2, Beckman Coulter).

### Immunohistochemistry

Fresh liver tissues were washed with PBS, fixed in 4% paraformaldehyde for 24 h, dehydrated with 70% ethyl alcohol, and embedded in paraffin. Liver tissues were then sectioned at 3 µm. Sections were de-paraffinized using xylene and ethanol. For antigen retrieval, slices were immersed in HistoVT One (Nacalai Tesque) at 37 °C for 20 min. Endogenous peroxidase activity was blocked using 3% H_2_O_2_ with methanol and DAKO wash buffer. Blocking One Histo (Nacalai Tesque) was added to samples for 20 min. F4/80 antibodies (HM1066, Hycult Biotechnology) with PBS were added and incubated overnight. The following day, biotinidase rat antibodies were applied. Staining was performed using the Vectastain Universal Elite ABC kit (Vector Laboratories Burlingame). All slides were counterstained with Mayer’s haematoxylin and mounted using permanent mounting media. The slides were analysed by fluorescence microscopy (BZ-X710; Keyence Japan).

### Measurements of in vivo endotoxin clearance

After resting for one day following exercise, all groups were injected in the tail vein with endotoxin at 0.01 µg/g body weight (E. coli O111:B4, Sigma-Aldrich). Blood samples were collected with heparin from the same vein at baseline (pre), and at 1.5, 3.0, 6.0, and 12.0 h following injection. Each sample was immediately centrifuged at 4000 rpm for 15 min. The supernatants were stored at −80 °C until assay. Endotoxin levels in plasma samples were assessed using the LAL pyrochrome reagent in Glucasheld buffer (Associates of Cape Cod). To remove the amber colour, we used the PyroColor Diazo Reagents kit (Associates of Cape Cod). Optical density was measured using an iMark Absorbance Microplate Reader at 540 nm (Bio Rad Laboratories).

### Measurements of cytokine and steroid hormone concentrations in peripheral blood

Prasma cytokine levels (TNF-α, IL-6, and IL-10) were measured using a multiplex bead analysis system (BioRad Laboratories). Serum testosterone (ADI-900-065, ENZO Life Sciences), estradiol (ES180S-100, Cal Biotech), corticosterone (501320, Cayman Chemical Company), DHEA (DI-900-093, ENZO Life Sciences), leptin (M1305, Morinaga Institute of Biological Science), and adiponectin (MRP300, R&D Systems) were measured according to the manufacturer’s instructions.

### *In vitro* experiments

Mouse RAW264.7 macrophage-derived cells were cultured in DMEM high glucose medium supplemented with 10% fetal bovine serum (FBS), penicillin (50 U/ml), and streptomycin (50 mg/ml) at 5% CO2, 95% humidity, 37 °C.

To evaluate foreign-body phagocytic activity, 2.5 × 10^5^ RAW264.7 cells were plated in 6-well plates and incubated for 24 hours. The cells were then incubated with DHEA (TCI) at various concentrations (1.0, 5.0, 10.0 ng/ml with DMSO) for 12 hours. Latex beads at 0.66 µl/ml DMEM were added for 12 hours. Following incubation, cells were fixed in 4% paraformaldehyde. For flow cytometric analysis, cells were washed with PBS on the dish and collected using a cell scraper. These samples were then washed twice by centrifugation at 4000 rpm for 3 min. Immediately thereafter, bead fluorescence in the cells was analysed by flow cytometry.

To evaluate inflammatory response to endotoxin, following 24-hour culture, the cells were incubated with DHEA (10.0 ng/ml with DMSO) for 12 hours prior to adding endotoxin (100 ng/ml) for 1 hour. After washing with PBS, the cells were lysed in SDS buffer, protease inhibitor cocktail, and phosphatase inhibitor cocktail (Nacalai Tesque). Lysates were stored at −80 °C until assay.

Protein concentration in cell lysates was measured using a BCA protein assay kit (Thermo Scientific Pierce). Dilution with lysis buffer was employed to normalize protein concentrations. Equal amounts of protein (10 µg per well) and corresponding loading buffer were mixed and analysed by gel electrophoresis. The proteins were transferred to nitrocellulose membranes and blocked with Blocking One-P (Nacalai Tesque) for 1 hour. β-Actin (SC-1616, Santa Cruz), IκB-α (SC-371, Santa Cruz), NFκB-p65 (C22B4, Cell Signaling Technology), and phospho-NFκB-p65 (93H1, Cell Signaling Technology) were diluted with Signal Enhancer HIKARI for Western Blotting and ELISA (Nacalai Tesque). The membranes were incubated at 4 °C overnight, followed by incubation with HRP-conjugated secondary antibodies for 2 hours at room temperature. Target proteins were visualized by Chemi-Lumi One Super (Nacalai Tesque) and then exposed using a ChemiDoc XRS+ system chemiluminescence imager (Bio-Rad). Image Lab software (Bio-Rad) was used for image acquisition and densitometry analysis.

### Statistical analysis

Statistical analysis was performed using SPSS Statistics for Macintosh, version 21.0 (IBM). Descriptive parameters are expressed as mean ± standard error (SE). For inter-group comparisons, non-parametric (Mann-Whitney U or Kruskal-Wallis test) tests were applied. Statistical significance was defined as *P* < 0.05 for all analyses.

## Results

### Changes in body weight and composition

**Figure 1:**
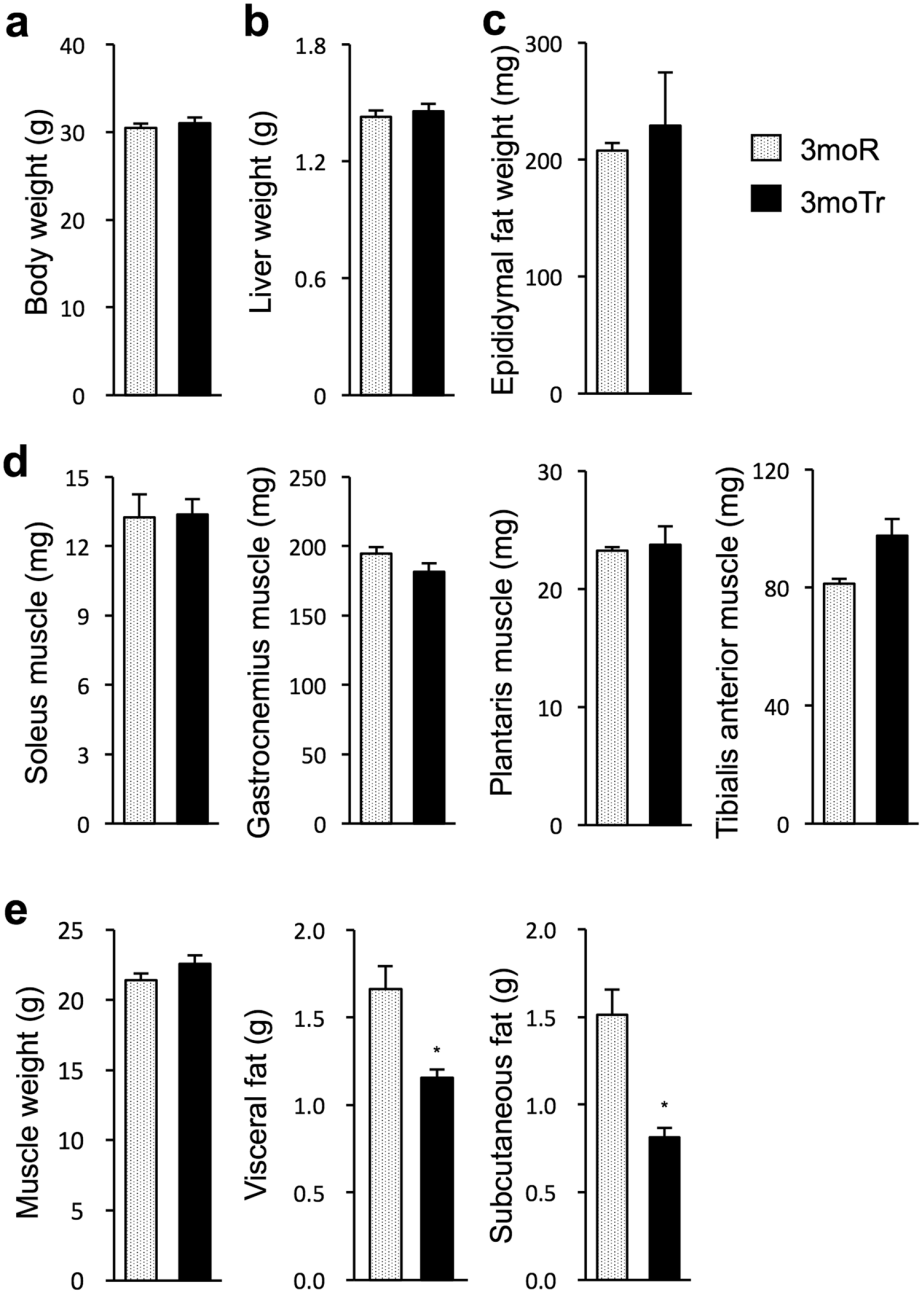
Changes in body weight and body composition. (**a**) Body weight. (**b**) Liver weight. (**c**) Epididymal fat weight. (**d**) Weight of individual skeletal muscles (soleus, gastrocnemius, plantaris, and tibialis anterior). (**e**) Weight of total-body skeletal muscle, visceral fat, and subcutaneous fat (analysed by CT for small animals). For all groups, n = 8. Values are expressed as mean ± standard error. *P < 0.05, significantly different from the 3moR group.

Figure 1a–d displays weights of body, liver, epididymal fat, and hind-limb skeletal muscle (soleus, gastrocnemius, plantaris, and tibialis anterior). At the 3-month time point, there was no significant difference in any parameter between the rest group (3moR) and the exercise group (3moTr). In the analysis by CT, there was no difference in the weight of total-body skeletal muscle between the 3moR and the 3moTr groups (Fig. 1e). Conversely, both the visceral fat mass and the subcutaneous fat mass were lower in the 3moTr group, compared with the 3moR group (Fig. 1e).

### Changes in number and foreign-body phagocytic capacity of Kupffer cells

**Figure 2:**
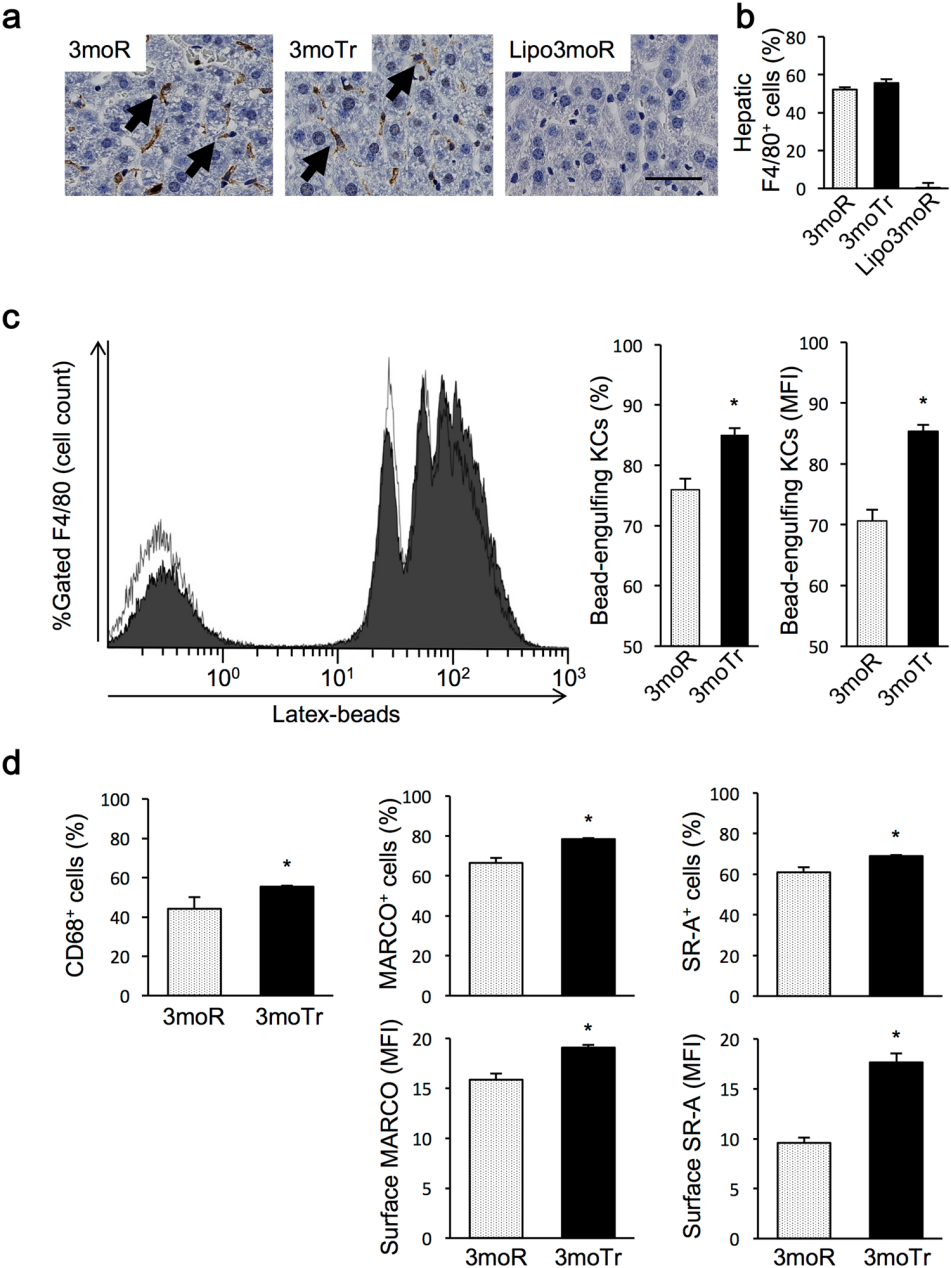
Changes in foreign-body phagocytic capacity and surface proteins of Kupffer cells (KCs). (**a**) Immunohistochemistry of F4/80 in liver. The arrow indicates the F4/80-positive cells. Scale bar, 50 µm. (**b**) Percentage of F4/80-positive cells in liver non-parenchymal cell fraction (flow cytometric analysis). (**c**) Latex beads phagocytic capacity of KCs. (**d**) Expression levels of surface proteins of KCs. For all groups, n = 8. Values are expressed as mean ± standard error. *P < 0.05, significantly different from the 3moR group.

Immunohistochemical staining of the livers revealed that there was no difference in the number of the KC surface marker F4/80-positive cells between the 3moR and the 3moTr groups. In the livers of the Lipo3moR mice, the number of F4/80-positive cells was decreased (Fig. 2a). Flow cytometric analysis of the hepatic non-parenchymal cell fraction revealed no difference in terms of percentage of F4/80-positive cells between the 3moR and the 3moTr groups. This percentage was lower in the Lipo3moR group (Fig. 2b).

Since KC number did not change following 3-month exercise training, foreign-body phagocytic capacity of KCs was analysed by flow cytometry (Fig. 2c). Uptake and fluorescence intensity of latex beads engulfed in F4/80-positive cells were determined (Fig. 2c). In the histogram, the fluorescence intensity of the right-side peak, representing the latex bead-engulfing cells, was increased in the 3moTr group. The number of KCs that had engulfed latex beads as a percentage of all KCs was significantly increased in the 3moTr group compared with the 3moR group. The mean fluorescence intensity (MFI) of the beads engulfed in KCs showed a significant difference in the 3moTr group as compared with that of the 3moR group. The number of CD68-positive KCs was significantly increased in the 3moTr group compared with the 3moR group (Fig. 2d). The percentage of MARCO-positive cells and the percentage of SR-A-positive cells were higher in the 3moTr group, compared with the 3moR group (Fig. 2d). Similarly, the MFI of these proteins were higher in the 3moTr group.

### Changes in in vivo endotoxin clearance and cytokine levels

**Figure 3:**
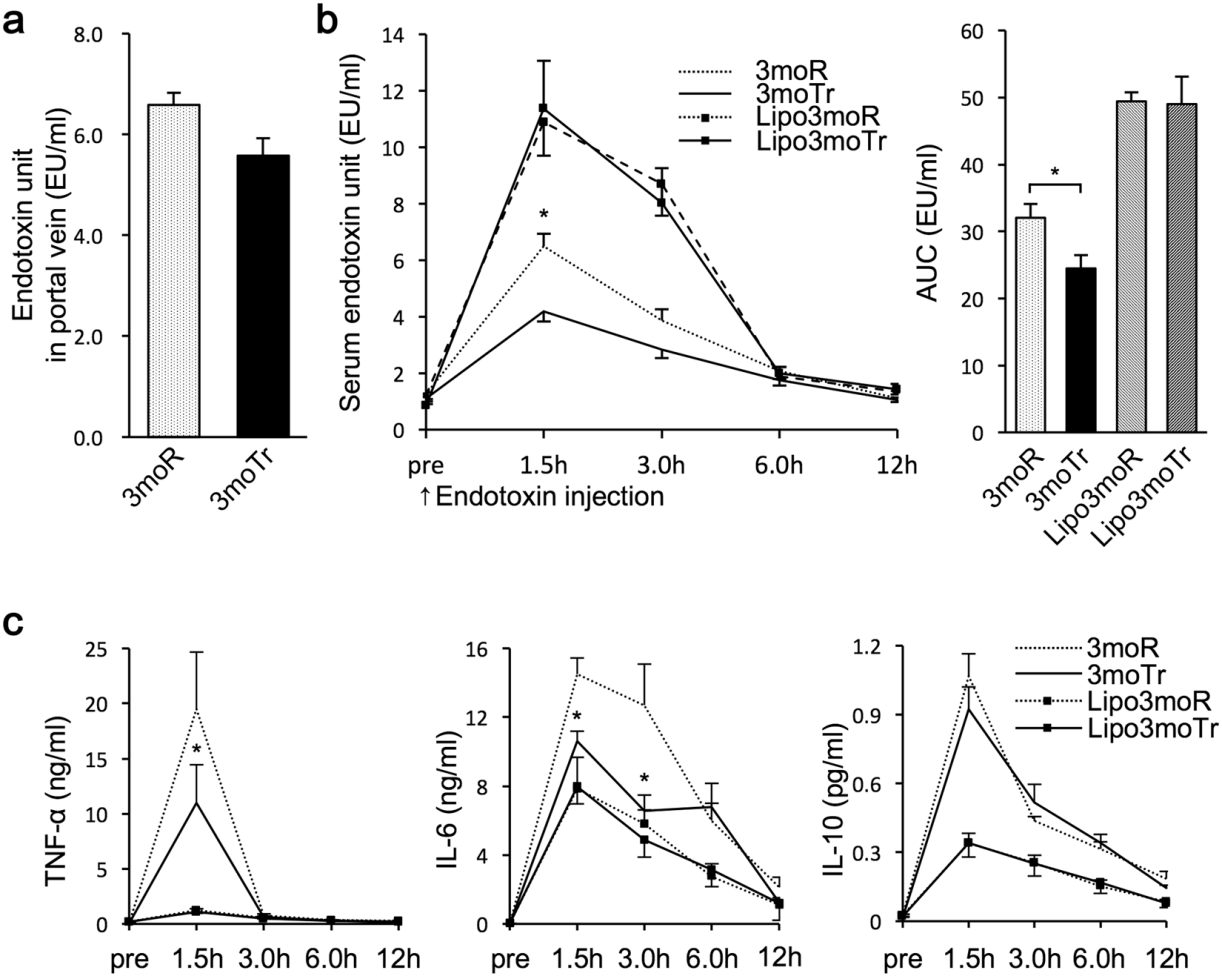
Changes in *in vivo* endotoxin clearance and cytokine levels. (**a**) Endotoxin levels in portal vein blood. Levels were determined immediately following the end of the final exercise load. (**b**) Time course of endotoxin levels in peripheral blood. (**c**) Time course of cytokine levels in peripheral blood. (**b**,**c**) After administration of endotoxin (0.01 µg/g body weight) into the tail vein, blood was sampled prior to administration (Pre), and at time points of 1.5 h, 3.0 h, 6.0 h, and 12 h following administration. For all groups, n = 8. Values are expressed as mean ± standard error. *P < 0.05, significantly different from the 3moR group.

Exercise training caused an increase in foreign-body phagocytic capacity of KCs (Fig. 2b). Endotoxin levels in portal vein blood did not change following 3-month exercise training (Fig. 3a). Therefore, to investigate the capacity of KCs to remove endotoxin, we assessed the time courses of endotoxin and cytokine levels in blood following the administration of endotoxin 24 hours following exercise training (Fig. 3b and c). In Fig. 3b, a peak appeared at 1.5 hours following endotoxin administration in the 3moR group, followed by a gradual decrease in endotoxin levels. In the 3moTr group, a peak in the endotoxin levels appeared at 1.5 hours, but the peak value was significantly lower. The peak values of endotoxin levels in the Lipo3moR and Lipo3moTr groups were higher than those of the 3moR and 3moTr groups. The area under the curve (AUC) value of the endotoxin levels was significantly lower in the 3moTr group compared with that of the 3moR group. The AUC values were higher in the Lipo3moR and Lipo3moTr groups. These results suggest that 3-month exercise training increased endotoxin clearance *in vivo*.

Since 3-month exercise training increased *in vivo* endotoxin clearance, changes in cytokine levels are shown in Fig. 3c. In the 3moR group, TNF-α and IL-6 levels peaked at 1.5 hours following endotoxin administration. This was followed by a gradual decrease. Similarly, in the 3moTr group, peaks appeared at the 1.5-hour time point, but the peak values of the TNF-α and IL-6 levels were significantly lower than those of the 3moR group. Cytokine levels in the Lipo3moR and Lipo3moTr groups were lower than those of the 3moR and 3moTr groups. These results suggest that 3-month exercise training reduced the inflammatory response to endotoxin. Plasma IL-10 levels peaked at 1.5 hours following endotoxin administration; however, exercise training had no effect on these levels. Following the macrophage depletion groups (Lipo3moR and Lipo3moTr), the peak values of the IL-10 levels were lower than those in the 3moR and 3moTr groups.

### Changes in blood steroid hormone levels and adipokine levels

**Figure 4:**
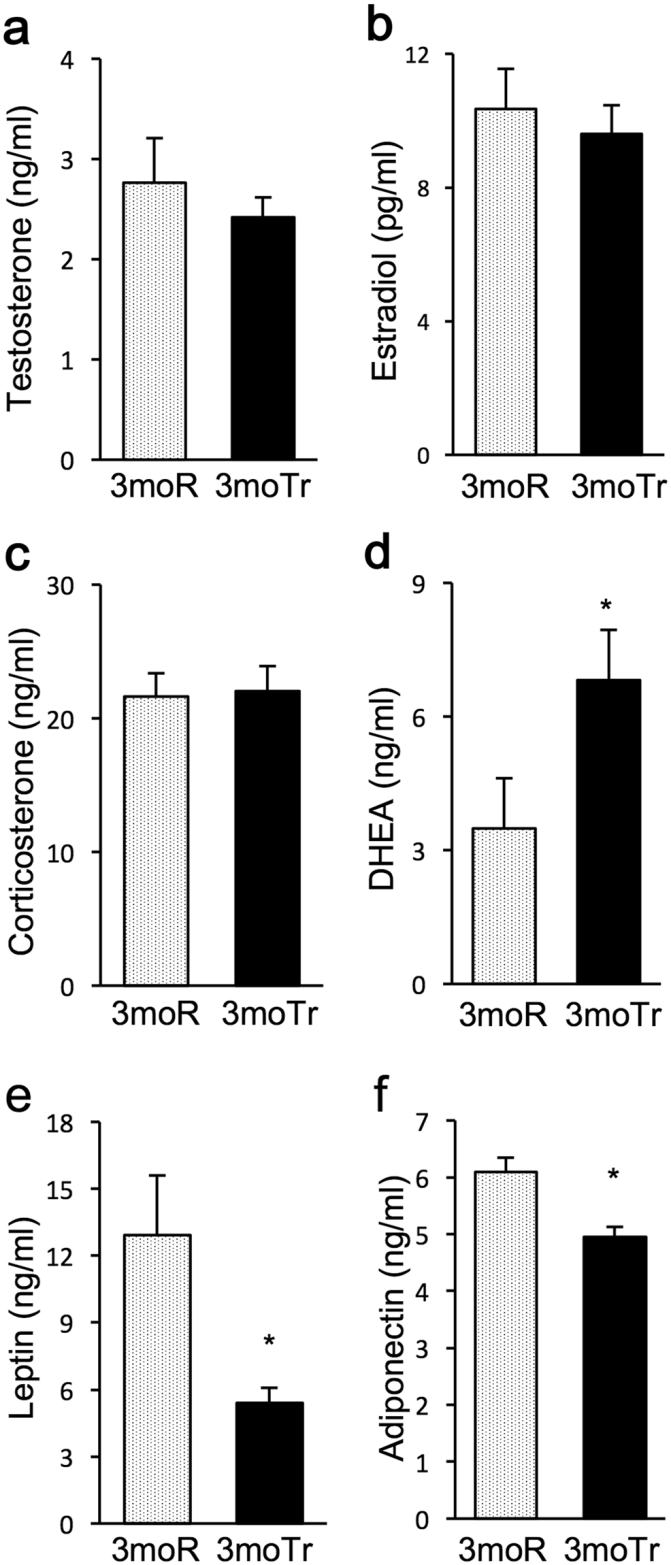
Changes in steroid hormone levels and adipokine levels in peripheral blood. (**a**) Testosterone. (**b**) Estradiol. (**c**) Corticosterone. (**d**) DHEA. (**e**) Leptin. (**f**) Adiponectin. For all groups, n = 8. Values are expressed as mean ± standard error. *P < 0.05, significantly different from the 3moR group.

Steroid hormones and adipokines have been reported to increase KC phagocytic capacity^18–22^. Therefore, we investigated exercise-induced changes in serum levels of steroid hormones and adipokines (Fig. 4). In all groups, the levels of testosterone, estradiol, and corticosterone showed no significant changes (Fig. 4a–c). DHEA levels were significantly higher in the 3moTr group compared with the 3moR group (Fig. 4d).

The levels of leptin, known as proinflammatory adipokine, were significantly lower in the 3moTr group compared with the 3moR group (Fig. 4e). Also, the levels of adiponectin, known as anti-inflammatory, were lower in the 3moTr group (Fig. 4f).

### DHEA-induced changes in foreign-body phagocytic capacity and the inflammatory response of macrophages to endotoxin

Three-month exercise training modified the KC phenotypes in association with increased DHEA levels in the blood (Fig. 4d). Following this observation, we investigated the effects of DHEA administration on the phenotypic changes of macrophage cells (RAW264.7) to endotoxin. The dose of DHEA administration was determined in reference to the differences in DHEA concentrations between the 3moR and the 3moTr groups (Fig. 4d).

**Figure 5:**
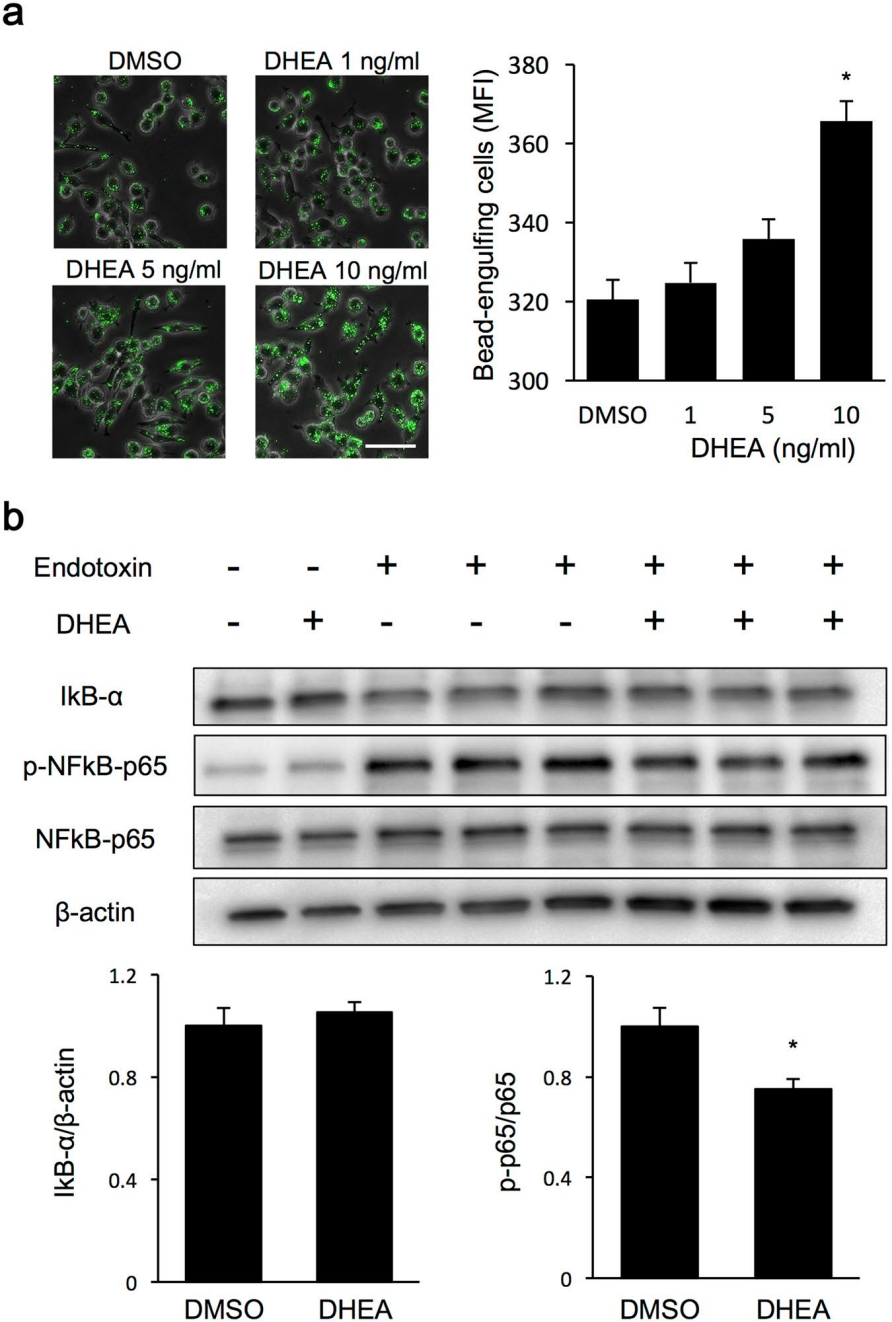
DHEA-induced changes in foreign-body phagocytic capacity and inflammatory response of macrophages to endotoxin. (**a**) Latex beads in RAW264.7 cells and their mean fluorescence intensity (MFI). Latex beads are shown in green. Scale bar, 50 µm. MFI of latex beads was determined by flow cytometry. (**b**) Endotoxin-induced changes in inflammatory response signal molecules. Analysis was performed 1 hour following the addition of endotoxin (100 µg/mL). For comparison of phagocytic capacity, n = 6 for all groups, and for comparison of inflammatory response, n = 3 for all groups. Values are expressed as mean ± standard error. *P < 0.05, significantly different from the DMSO group.

The addition of DHEA to RAW264.7 significantly increased the MFI of the cells that had engulfed beads at a DHEA concentration of 10 ng/mL compared with control. DHEA increased bead fluorescence intensity in a concentration-dependent manner. An increase in the number of beads could also be confirmed by flow cytometry (Fig. 5a). Endotoxin administration (100 ng/mL) accelerated the phosphorylation of NFκB-p65 and degradation of IκB-α. Following the addition of DHEA (10 ng/mL), there was no difference in degradation of IκB-α, but phosphorylation of NFκB-p65 was significantly attenuated, compared with the group without the addition of DHEA (Fig. 5b﻿ and Fig. S1).

## Discussion

The principal findings of this study are as follows: exercise training for 3 months caused no change in portal vein endotoxin levels or KC number; exercise training caused an increase in foreign-body phagocytic capacity and an increase in the *in vivo* clearance of endotoxin injected exogenously; exercise training triggered an attenuation in endotoxin-induced inflammatory responses and thereby in inflammatory cytokine levels. The observed effects of exercise training disappeared in KC-depleted mice, suggesting that the observed effects were caused by exercise-induced changes in KC function.

Exercise training caused an increase in the number of beads engulfed by KCs and a potentiation of *in vivo* endotoxin clearance (Figs 2c and 3b), without altering endotoxin levels in portal vein blood (Fig. 3a). In a report on the relationship between KC phagocytic capacity and exercise, Yano *et al*. reported that in F344 rats, acute exercise caused endotoxin level increases in portal vein blood, followed by intestinal mucosal damages, and a consequent increase in KC phagocytic capacity^16^. In isolated KC experiments, endotoxin increased phagocytic capacity^23^. In our study, exercise training for 3 months caused an increase in KC phagocytic capacity and a potentiation of *in vivo* endotoxin clearance (Figs 2c and 3b), without alteration of endotoxin levels in portal vein blood (Fig. 3a). The molecular mechanisms mediating these effects are not currently understood. In another report, when [^3^H]-endotoxin was injected, approximately 80% was detected in the liver^24^. Therefore, phagocytic capacity of KC is suspected to be important. Taken together, these results suggest that the effects of exercise training on endotoxin clearance and inflammatory response may be attributable to modification of KC function. To the best of our knowledge, our study provides the first evidence of the benefits of exercise training on KC function.

Exercise training for 3 months increased initial bead uptake by KCs (at 5 min following administration) (Fig. 2c). An increase in CD68 and scavenger receptors on the surface of KCs may contribute to increased phagocytic capacity (Fig. 2d). The CD68-positive cells among KCs constitute the cell population with strong bead phagocytic capacity^23^. Scavenger receptors (MARCO, SR-A) are involved in phagocytic capacity and endotoxin clearance^25,26^. Exercise training increased expression of these surface molecules on KCs, resulting in an increase in KC phagocytic capacity. We suggest that this may cause lower peak endotoxin levels in peripheral blood following endotoxin administration. One study reports that acute exercise greater than 30 min contributes to an increase in carbon clearance^27^. This effect may support our results regarding the exercise-induced increase in foreign-body phagocytic capacity of KCs.

We investigated the effects of exercise training on the *in vivo* inflammatory response to endotoxin. Following endotoxin administration, peripheral blood levels of inflammatory cytokines such as TNF-α and IL-6, and the level of the anti-inflammatory cytokine IL-10 achieved their peak values at 1.5 h (Fig. 3c). Exercise training decreased the inflammatory response to endotoxin via attenuation of the increase in peak values of TNF-α and IL-6 levels at 1.5 h (Fig. 3c). Increased KC phagocytic capacity due to 3-month exercise training resulted in rapid clearance of endotoxin from the body, leading to an attenuation of the inflammatory response. However, no differences between the exercise and rest groups were observed with respect to IL-10 levels (Fig. 3c). Ortega *et al*. reported that in human monocytes following aquatic exercise training, the amount of IL-10 secreted following endotoxin administration was higher in the exercise group than in the rest group^28^. More detailed investigations of this phenomenon are required in the future.

We investigated the mechanisms of exercise-induced alteration in KC function in the absence of increases in portal vein blood endotoxin levels. We measured peripheral blood levels of steroid hormones, which have been reported to affect foreign-body phagocytic capacity^18–20^. Following 3-month exercise training, DHEA levels alone were increased. This coincides with the findings of another mouse study, in which endotoxin was injected following DHEA administration. In that study, the survival rate of mice was increased and secretion of TNF-α was inhibited simulatneously^29^. These results suggest that exercise training may increase blood DHEA levels to reduce the inflammatory response of KCs to endotoxin stimulation.

We investigated DHEA-induced changes in KC function in *in vitro* experiments. The addition of DHEA to RAW264.7 cells, at a concentration close to the peripheral blood levels after 3-month exercise training (Fig. 4d), caused a dose-dependent increase in latex bead phagocytic capacity (Fig. 5a), and inhibition of endotoxin-induced phosphorylation of NFκB-p65 (Fig. 5b). These *in vitro* results suggest that the increase in blood DHEA levels by 3-month exercise training may induce an increase in foreign-body phagocytic capacity and a decrease in the inflammatory response of KCs to endotoxin. In a previous study, it was reported that the addition of EPI, a DHEA analogue, to human monocytes increased phagocytosis of malaria-infected erythrocytes (ring stage)^30^. Another study showed that the addition of DHEA to RAW264.7 inhibited endotoxin-induced TNF-α secretion^31^. These reports may support the results observed in our study.

It has been reported that adipokines can increase Kupffer cell phagocytic capacity^21,22^; thus, leptin, a proinflammatory adipokine, and adiponectin, which is anti-inflammatory, increase macrophage phagocytic capacity. In this study, blood leptin and adiponectin levels were both lower in the 3moTr group than in the 3moR group (Fig. 2b). These results were consistent with those of earlier studies^32,33^. Thus, it cannot be inferred that increased adipokine levels affect the increase in Kupffer cell phagocytic capacity induced by the 3-month exercise training.

Patients with NAFLD suffer various symptoms, including elevated blood endotoxin levels^4^, decreased KC phagocytic capacity^9^, and enhanced inflammatory responses^34^. Hepatic pathology such as fat accumulation and fibrogenesis in NAFLD correlate negatively with blood DHEA levels^35^. In our recent report^15^, we demonstrated that exercise training attenuates NAFLD lesions simultaneously with increasing KC phagocytic capacity and decreasing the immune inflammatory response. This study points to one potential molecular mechanism by which exercise training may be useful for the management of NAFLD.

## Conclusion

Exercise training for 3 months in mice increased KC foreign-body phagocytic capacity, thereby increasing *in vivo* endotoxin clearance, and attenuating the increase in inflammatory responses to endotoxin. It is likely that exercise-induced increases in DHEA production may be associated with these observations.

## Electronic supplementary material

Supplementary information
