# Supplementary material for: Exercise training enhances in vivo clearance of endotoxin and attenuates inflammatory responses by potentiating Kupffer cell phagocytosis
Source: Sci Rep. 2017 Sep 20;7:11977. doi: 10.1038/s41598-017-12358-8 (PMC5607327; doi:10.1038/s41598-017-12358-8)
Supplement: Supplementary file 1 — Supplementary information [file 41598_2017_12358_MOESM1_ESM.pdf]

Supplementary information

Exercise training enhances *in vivo* clearance of endotoxin and attenuates inflammatory responses by potentiating Kupffer cell phagocytosis

Shoichi Komine[1,2], Kentaro Akiyama[1,3], Eiji Warabi[4], Sechang Oh[2,5], Keisuke Kuga[6], Kazunori Ishige[7], Shinji Togashi[8,†], Toru Yanagawa[9], and Junichi Shoda[2,5,*]

[1]Graduate School of Comprehensive Human Sciences, University of Tsukuba, Ibaraki, 305-8575, Japan;

[2]The Center of Sports Medicine and Health Sciences, Tsukuba University Hospital, Ibaraki, 305-8576, Japan;

[3]Japan Society for the Promotion of Science, Tokyo, 102-0083, Japan;

[4]Division of Molecular and Cellular Physiology, Faculty of Medicine, University of Tsukuba, Tsukuba, Ibaraki, 305-8575, Japan;

[5]Medical Sciences, Faculty of Medicine, University of Tsukuba, Tsukuba, Ibaraki, 305-8575, Japan;

[6]Division of Cardiology, Faculty of Medicine, University of Tsukuba, Tsukuba, Ibaraki, 305-8575, Japan;

[7]Division of Gastroenterology, Faculty of Medicine, University of Tsukuba, Tsukuba, Ibaraki, 305-8575, Japan;

[8]Division of Plastic Surgery, Faculty of Medicine, University of Tsukuba, Tsukuba, Ibaraki, 305-8575, Japan;

[9]Division of Oral and Maxillofacial Surgery, Faculty of Medicine, University of Tsukuba, Tsukuba, Ibaraki, 305-8575, Japan;

[†]The present affiliation: Plastic Surgery, Shonai Amarume Hospital, Yamagata, 999-7782, Japan;

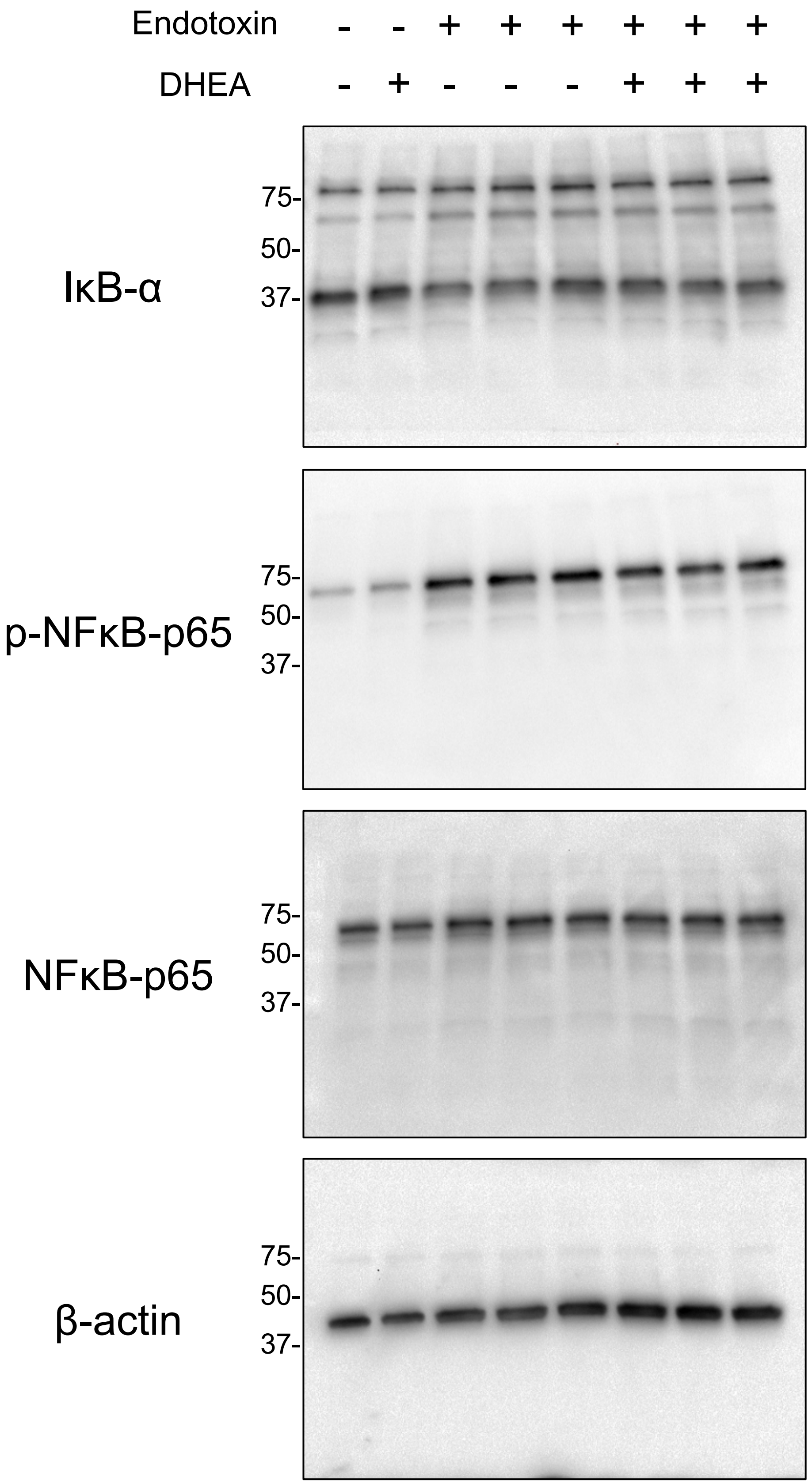

Supplementary figure S1. Full-length images of figure 5b.

The origin of these panels is identical with figure 5b showing western blotting with IκB-α, phospho-NfκBp65, NfκBp65, and β-actin antibody.
